# *Coxiellaburnetii* (Q fever) exposure in wildlife professionals

**DOI:** 10.3389/fpubh.2024.1466981

**Published:** 2024-11-13

**Authors:** Danilo Alves de França, Louise Bach Kmetiuk, Orlei José Domingues Rodrigues, Giovanni Augusto Kalempa Panazzolo, Vivien Midori Morikawa, Ana Íris de Lima Duré, Helio Langoni, Giovani Marino Fávero, Alexander Welker Biondo

**Affiliations:** ^1^Department of Animal Production and Preventive Veterinary Medicine, School of Veterinary Medicine and Animals Science, São Paulo State University, Botucatu, Brazil; ^2^Zoonoses Surveillance Unit, City Secretary of Health, Curitiba, Brazil; ^3^Graduate College of Pharmaceutical Sciences, State University of Ponta Grossa, Ponta Grossa, Brazil; ^4^City Secretary of Environment, Curitiba, Brazil; ^5^Service of Virology and Rickettsiosis, Octavio Magalhaes Institute Ezequiel Dias Foundation, Belo Horizonte, Brazil; ^6^Department of Veterinary Medicine, Federal University of Paraná, Curitiba, Brazil; ^7^Department of Comparative Pathobiology, Purdue University, West Lafayette, IN, United States

**Keywords:** one health, Q fever, zoo workers, zoonoses, wildlife

## Abstract

**Introduction:**

Although occupational exposure to *Coxiella burnetii* has been studied previously, the zoonotic risk in wildlife environments remains unclear and has yet to be fully established.

**Methods:**

Accordingly, the present study aimed to serologically assess professionals with daily contact with free-living and captive wildlife in Paraná State, Brazil, along with the potential associated risk factors for *C. burnetii* exposure.

**Results:**

Overall, 25 out of 309 (8.1%) wildlife professionals were seropositive, including 6/54 (11.1%) national and 7/125 (5.6%) state park employees, 6/92 (6.5%) zookeepers, and 6/38 (15.8%) animal service workers, with titers ranging from 32 to 128. No statistical association was found between seropositivity and associated risk factors, including the working location.

**Discussion:**

Our results differ from those of previous studies in Brazil, which found 8/893 (0.9%) indigenous, 1/18 (5.5%) police officers, and 44/200 (22.0%) former Black slaves to be seropositive. This study is the first serological investigation of *C. burnetii* among park rangers, zookeepers, and animal service workers in Brazil, showing no statistically significant risk factors for seropositivity. As the seroprevalence in this study was higher than that in previous surveys of healthy (asymptomatic) human populations, *C. burnetii* exposure may also be an occupational risk for wildlife professionals owing to their contact with the natural environment in Brazil.

## 1 Introduction

Q fever, a disease caused by *Coxiella burnetii* bacteria, is typically described as an asymptomatic infection; however, symptoms may arise over time due to an acute self-limiting infection or a chronic and persistent infection with potentially lethal outcomes (1). By persisting inside phagolysosomal cells over time, *C. burnetii* is associated with vascular injury in cardiac patients (2). Infection primarily occurs as a consequence of contact with an infected animal, inhalation of contaminated aerosols, or, to a lesser degree, consumption of contaminated raw milk, contact with fomites, and tick bites (1). The transmission route and individual susceptibility affect the infectious dose and subsequent disease progression (3).

Domestic ruminants, including goats, sheep, and cattle, have been well established as playing an important role in the *C. burnetii* zoonotic cycle, primarily due to high levels of human contact compared to other livestock species (4, 5). In addition to ruminants, several other mammalian species, particularly rodents, birds, and reptiles, may act as reservoirs, with natural *C. burnetii* infections already described in more than 100 wildlife species (6, 7). Although the small sample sizes of such studies strongly limit clinical conclusions, some ruminants and sea lions have presented symptoms of reproductive failure (8).

Q fever remains a globally underestimated disease, particularly in developing countries where surveillance and reporting systems may be limited (9). It is classified as a notifiable disease in many regions, including the EU/EEA and other countries such as Australia, where public health authorities require reporting of confirmed cases (10). However, despite this notifiable status, subnotification is a persistent challenge. Although outbreaks of Q fever are relatively rare, they can occur, especially in areas with high concentrations of livestock, such as farms or rural communities. One of the most significant outbreaks took place in the Netherlands between 2007 and 2010, resulting in over 4,000 human cases (11). Although Q fever is not considered a notifiable disease worldwide (12), information on this disease in Europe can be obtained at the European Center for Disease Control (13). In addition, *C. burnetii* exposure has been increasingly reported in the Americas, including Brazil, through serological surveys and retrospective hospital studies (1). Brazilian indigenous populations have shown a significantly higher frequency of *C. burnetii* in communities located in natural forests with hunting practices (14). Interestingly, hunting dogs from Australian Aboriginal villages are more likely to be seropositive than house and shelter dogs (15).

Wildlife is home to countless microorganisms that may evade or overcome the human immune response (16). In addition, the invasion, fragmentation, and deforestation of natural forest areas—aggravated by ecological imbalances and climate change—may lead to increased overlap among humans, livestock, wildlife, and their vectors, thus heightening the likelihood of disease exposure, infection, and transmission (16). Studies have shown exposure to *C. burnetii* among professionals in direct contact with wildlife, such as wildlife rehabilitators in Australia (17), forestry workers in Poland (18) and Italy (19), and veterinarians worldwide (20–25). Although occupational exposure to *C. burnetii* has been studied, the zoonotic risk in wildlife environments remains unclear and needs further establishment. Accordingly, the present study aimed to serologically assess professionals with daily contact with free-living and captive wildlife for *C. burnetii*, along with potential associated risk factors.

## 2 Materials and methods

### 2.1 Ethics statement

This study was approved by the Ethics Committee on Human Health of the Brazilian Ministry of Health (protocol: 97639017.7.0000.0102). Questionnaires and sampling were officially included in the activities of all participating institutions.

### 2.2 Study design and sample collection

**Figure 1:**
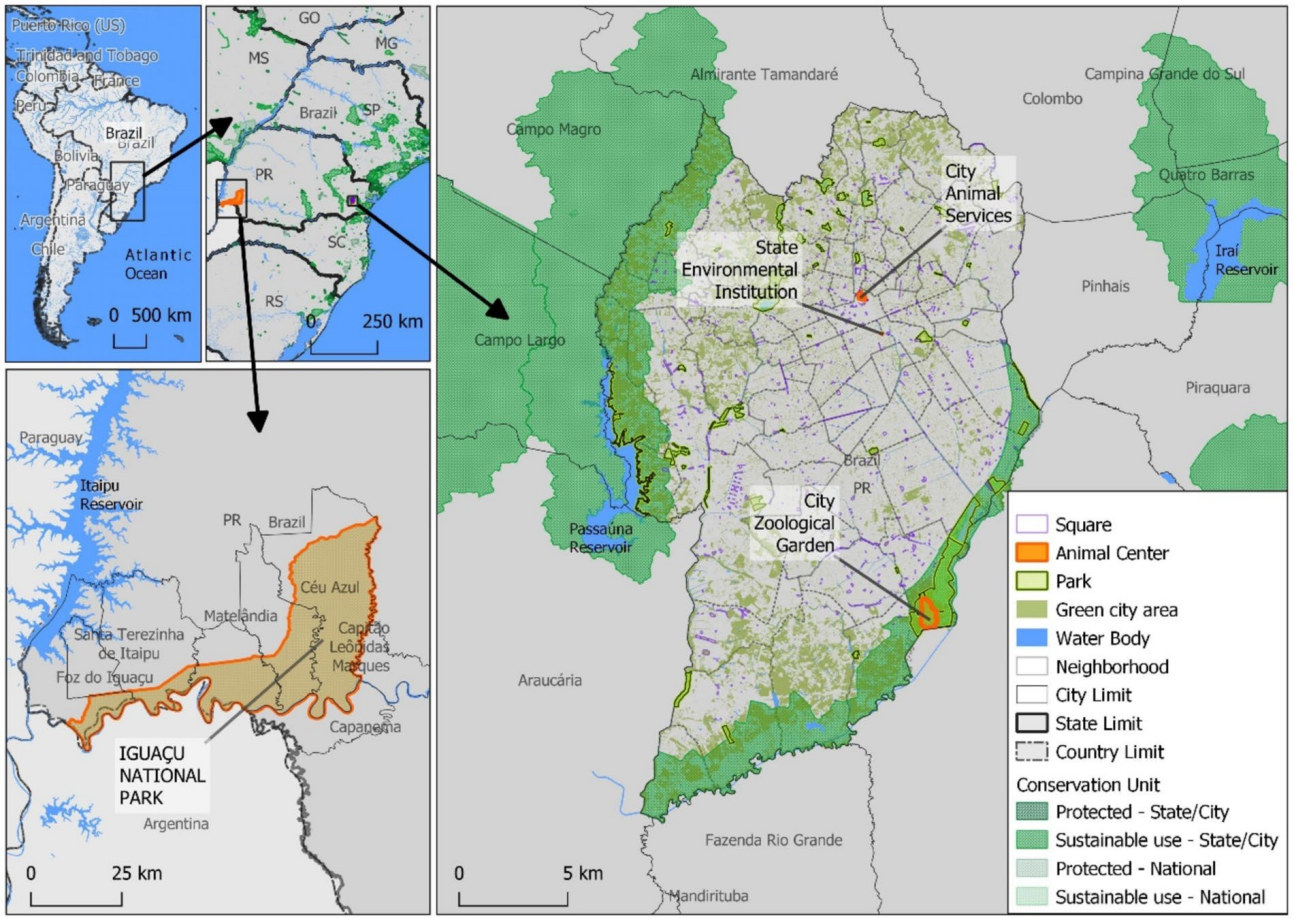
Sampling locations of wildlife professionals from Paraná State, southern Brazil.

The four governmental institutions surveyed in this study were responsible for handling domestic and wildlife animals in city, state, and federal areas within Paraná State, with headquarters in two major Brazilian cities approximately 635 km (395 miles) apart: Curitiba, the capital of Paraná State, and Foz do Iguaçu, a tri-border city along the junction of Brazil, Argentina, and Paraguay (Figure 1). All professionals had contact with domestic and wildlife animals to varying degrees as part of their daily duties, working within the Brazilian Atlantic Forest biome of Paraná state, southern Brazil, and came into contact with various wildlife species, including mammals, birds, amphibians, and reptiles.

Figure elaborated by the authors with QGIS 3.18. Direct link to the source of Icons and symbols used: https://github.com/qgis/QGIS/tree/master/images/svg; Direct link of the boundaries from Brazilian government official public data, used as background base layer: https://geoftp.ibge.gov.br/cartas_e_mapas/bases_cartograficas_continuas/bc250/versao2021/post_gis/bc250_2021_11_18.zip; Conservation Units data from ICMBio https://www.gov.br/icmbio/pt-br/assuntos/dados_geoespaciais; Neighborhood, Green Areas, Parks and animal center data from Curitiba https://ippuc.org.br/geodownloads/geo.htm; Country boundaries from WorldBank https://datacatalog.worldbank.org/search/dataset/0038272/World-Bank-Official-Boundaries.

The major City Animal Services and City Zoo were located in Curitiba (25° 25′ 47″ S; 49° 16′ 19″ W), the capital of Paraná State, with an area of 432 km^2^ (166.8 square miles). It is ranked 8th in population nationally, with 1.8 million inhabitants, 5th in gross domestic product, and 10th in the human development index, with a score of 0.823 (very high), out of 5,570 municipalities in Brazil. Curitiba has long been considered the most environmentally sustainable city in Brazil, with over 50 city parks and preservation areas within city limits. In addition, the State Environmental Institute had its headquarters in Curitiba, responsible at the time for the management of 70 conservation units, totaling 12,502.24 km^2^ (4,827.2 square miles) of preserved areas, approximately 28 times the size of Curitiba and 16 times the size of New York City.

The other City Animal Services was located in Foz do Iguaçu (25° 32′ 49” S; 54° 35′ 11” W), a far-western city of Paraná State, with an area of 609.19 km^2^ (235.21 square miles), located in the tri-border area of Brazil, Argentina, and Paraguay. Foz do Iguaçu is considered one of the top five tourist destinations in Brazil. At the time, it is ranked 97th in population, with 258,532 inhabitants (top 1.7%), 59th in gross domestic product (top 1.1%), and 526th in human development index, with a score of 0.751 (high) (top 9.4%), out of 5,568 Brazilian municipalities. In addition, Iguaçu National Park, with headquarters in Foz do Iguaçu, spreads over 13 other municipalities, totaling 1,852.62 km^2^ (715.30 square miles), with the longest stretch being 420 km (261.0 miles) (Table 1).

**Table 1:** Sampling locations of wildlife professionals, with their corresponding number of samples and administrative unit level.

Location	Institution	Level	Sampled professionals
	City Animal Services	City	38
Curitiba	City Zoological Garden	City	92
	State Environmental Institution	State	125
Foz do Iguaçu	Iguaçu National Park	Federal	54
		Total	309

Blood samples were collected after participants voluntarily signed a consent form at the headquarters of each institution, and an epidemiological questionnaire was administered. A total of 10 mL of blood was collected from each participant by cephalic puncture, performed by accredited physicians and nurses. The samples were placed in tubes without anticoagulant, centrifuged at 800 g for 5 min, and the serum was separated and stored at-20°C until processing.

### 2.3 Serological analysis

An in-house immunofluorescence test was performed according to the manufacturer’s protocol, which was previously developed and validated at the Brazilian Reference Laboratory (Ezequiel Dias Foundation, Belo Horizonte, MG, Brazil) (26). This assay is based on *Coxiella burnetii* antigen from *Amblyomma tigrinum* ticks, Argentina strain At12 (ST-73) (27), produced in embryonated eggs. Positive and negative controls, obtained from previously tested human samples during routine laboratory procedures, were placed into slide wells containing the antigens (30 μL), along with 1:64 diluted serum aliquots in phosphate-buffered saline (0.1 M; pH 7.2). Slides were then incubated at 37°C for 30 min, washed with phosphate-buffered saline, and dried in a moist chamber. A 30 μL volume of fluorescein isothiocyanate-anti-IgM antibody was added to the wells, followed by another incubation in a moist chamber at 37°C for 30 min (26). The reactions were observed under a commercial immunofluorescence microscope (Olympus BX53; Photonic Solutions Inc., Mississauga, ON, Canada) equipped with a 40x objective lens. For each slide, positive and negative controls were prepared using samples from patients previously diagnosed in our laboratory. Samples were initially diluted to 1:32 according to the standard protocol, with the volume adjusted to fit into the dilution plate wells, and a cutoff point of 1:64 was used. Positive samples were serially diluted until a final titer was reached. An IgG titer of 1:64 has been considered adequate for epidemiological investigations, as cross-reactivity with *Bartonella* and *Legionella* species typically occurs with lower titers and only with IgM antibodies (28).

### 2.4 Statistical analysis

Data from the epidemiological questionnaires were initially grouped to investigate the risk factors associated with seropositivity. Univariate and multivariate analyses were performed using Pearson’s chi-square test. The odds ratios and 95% confidence intervals were calculated using the coefficients obtained for each predictor variable. The most appropriate model was chosen based on the variables that showed significant associations (*p* < 0.05). All tests were performed using SAS Studio 3.81 (SAS Institute Inc., Cary, NC, United States).

## 3 Results

Overall, 25 out of 309 (8.09%; 95% confidence interval: 5.54–11.67) wildlife professionals were seropositive for *C. burnetii*, including 6/54 (11.1%) national and 7/125 (5.6%) state park employees and 6/92 (6.5%) zookeepers and 6/38 (15.8%) animal service workers, with titers ranging from 32 to 128. Two zoo professionals in this study reached titers of 1:128, whereas the other seropositive professionals had titers of 1:64. Variables that were statistically associated with seropositivity included sex, age, job position, forest access, forest access frequency, tick bites, consumption of raw or undercooked meat, and contact with abortion remains (Table 2). However, owing to the low *C. burnetii* seropositivity and limited statistical power, the associated risk factors could not be thoroughly assessed. No statistically significant associations were found between *C. burnetii* seropositivity and sex (*p* = 0.4440), age (*p* = 0.7636), job position (*p* = 0.3373), forest access (*p* = 0.4685), forest access frequency (*p* = 0.7472), tick bites (*p* = 0.7106), consumption of raw or undercooked meat (*p* = 0.1880), or contact with abortion remains (*p* = 0.9838).

**Table 2:** Associated risk factors of *Coxiella burnetii* exposure in wildlife professionals, Paraná State, southern Brazil.

Variables	*C. burnetii* positive	*C. burnetii* negative	OR (95% CI)	*p-value*	Total population
Gender				0.4440	
Female	11	142	1.0 (ref)		153
Male	15	141	0.73 (0.32–1.64)		156
Age				0.7636	
18 to 30 years old	6	97	1.0 (ref)		103
31 to 40 years old	9	71	0.49 (0.17–1.43)		80
41 to 50 years old	5	54	0.67 (0.19–2.29)		59
51 to 60 years old	4	48	0.74 (0.20–2.76)		52
> 60 years old	1	14	0.87 (0.10–7.74)		15
Job position				0.3373	
Veterinarian	1	20	1.0 (ref)		21
Biologist	3	19	0.32 (0.03–3.32)		22
Endemic control agent	6	28	0.23 (0.03–2.09)		34
Environmental engineer	1	18	0.90 (0.05–15.5)		19
General services	5	64	0.64 (0.07–5.80)		69
Management	3	22	0.37 (0.04–3.82)		25
Technical	4	75	0.94 (0.10–8.86)		79
Trainee	2	38	0.95 (0.08–11.1)		40
Forest area access				0.4685	
Yes	17	212	1.0 (ref)		229
No	8	72	0.72 (0.30–1.74)		80
Frequency of forest area access				0.7472	
None	8	71	1.0 (ref)		79
Less than once a month	4	44	1.23 (0.35–4.36)		48
Less than once a week	7	64	1.03 (0.35–3.00)		71
More than once a week	2	44	2.47 (0.50–12.2)		46
Everyday	4	61	1.72 (0.49–5.99)		65
Tick bites				0.7106	
Yes	7	70	1.0 (ref)		77
No	18	214	1.19 (0.48–2.96)		232
Intake of raw or undercooked meat				0.1880	
Yes	14	120	1.0 (ref)		134
No	11	164	1.73 (0.76–3.97)		175
Contact with abortion remains				0.9838	
Yes	4	45	1.0 (ref)		49
No	21	239	1.01 (0.33–3.09)		260

*p*- value < 0.05 indicates statistical difference within the categories. 1.0 (ref.): reference category. OR (95% CI): odds ratio (95% confidence interval).

## 4 Discussion

This study presents the first serological investigation of *C. burnetii* in wildlife professionals in Brazil, including park rangers, zookeepers, and animal service workers. Wildlife may serve as a source of exposure for professionals worldwide, as *C. burnetii* has been detected in tissue samples of 5.1% of roe deer (*Capreolus capreolus*), 4.3% of wild boars (*Sus scrofa*), 9.1% of European hares (*Lepus europaeus*), 11% of vultures (*Gyps fulvus*), and 14% of black kites (*Milvus migrans*) in northern Spain (29). In addition, a study in a Q fever-endemic area of Cyprus detected *C. burnetii* in mouflons (23/74), foxes (9/32), hares (15/31), and birds (41/131), with 56/195 (28.9%) infected ticks (30) and molecular detection in 12% of rat fleas and 47.6% of fox fleas (31). Although only a few studies of *C. burnetii* have been conducted in Brazil, molecular detection has been reported in 6/131 (4.6%) wild rodents in southeastern Brazil (32), 4/21 (19.0%) non-hematophagous bats in northeastern Brazil (28), and 9/169 (5.32%) deer in southeastern and central-western Brazil (33).

Studies on *C. burnetii* exposure among wildlife professionals have been conducted worldwide (17–19, 34). In Australia, 9/147 (6.1%) unvaccinated wildlife rehabilitators were seropositive, and Q fever vaccination was recommended because of its endemicity in this country (17). In Italy, 5/181 (2.8%) forestry rangers with a recent history of tick bites presented antibodies against *C. burnetii* (19). In Poland, 14/216 (6.4%) employees of National Forests were seropositive, suggesting high contact with *C. burnetii*-infected ticks in the study area (18). Active forestry workers showed a higher rate of previous exposure to *C. burnetii* (13/202; 6.4%) compared with that of supervisory forestry staff and muskrat catchers (4/110; 3.6%) in the Netherlands (34). Despite the limited sample size, national park employees (6/54; 11.1%) and animal service workers (6/38; 15.8%) showed higher seropositivity for *C. burnetii*, which may be associated with spending more time outdoors in wildlife environments and/or direct contact with animals, compared with that of state park employees (7/125; 5.6%) and zookeepers (6/92; 6.5%). Thus, further studies should be conducted with larger sample sizes and different populations of wildlife professionals to fully establish the impact of *C. burnetii* as an occupational risk associated with wildlife environments worldwide.

Given that human seroprevalence of *C. burnetii* in most countries has been reported to be low (12), this study revealed a higher overall seropositivity, which may have resulted from contact with wildlife. Nonetheless, the routes of transmission and elimination in wildlife remain unclear, as *C. burnetii* may behave differently depending on the host animal species, a topic that has not been widely studied to date.

Recent surveys of asymptomatic human populations in Brazil showed seroprevalence rates ranging from 3.2 to 4.5% (1, 35), as well as rates of 1/18 (5.5%) police officers, 8/893 (0.9%) indigenous individuals, and 44/200 (22.0%) quilombola individuals in Paraná State, most of whom live in rural areas near cattle farms (14, 36, 37). Although the relatively high seropositivity observed in the present study suggests that wildlife is an alternative source of human infection, no statistically significant associated risk factors were confirmed, including job position. Given that previous Brazilian studies with higher seropositivity involved individuals at occupational risk, further studies are needed to fully establish the role of wildlife in human infections.

Although this was a prevalence study in asymptomatic human populations, these individuals may develop persistent infections, and *C. burnetii* can be reactivated under immunosuppressive conditions. Serious complications, such as endocarditis, hepatitis, and meningitis, may occur (2, 38, 39), with one patient with Q fever in Brazil presenting with thrombocytosis and a 40-d fever (40).

Samples from zoo workers were collected at the end of the COVID-19 pandemic using epidemiological questionnaires focused on febrile symptoms. Individuals presenting with flu-like symptoms mostly tested negative for COVID-19. Of the 6/92 (6.5%) *C. burnetiid*-seropositive individuals, five experienced flu-like symptoms similar to Q fever, and four tested negative for COVID-19. Because such data were not available for other locations, no statistical analysis could be performed.

General preventive measures against *C. burnetii* infection have mostly focused on animal transmission. These measures include avoiding contact with animals, particularly during delivery and birth, even if the animals appear healthy, and avoiding the consumption of raw milk or raw milk products (41). For individuals in contact with wildlife, the infection risk may be effectively reduced through a set of measures, such as vaccination (which is not currently available in Brazil), proper manure and shearing management, isolation of breeding areas when sick animals are present, proper disposal of risk materials, limiting visitor and unsafe contact, controlling domestic and wild mammal reservoirs, and tick control (42). In addition, effective preventive measures may require continuous passive and active surveillance through a unified and accessible database shared by public health and veterinary services. This should be complemented by environmental and worker health monitoring, along with oversight of wildlife-livestock-periurban interfaces and relevant organizations. Such measures would assist in the prompt identification of this and other reemerging and novel pathogens, aiding in outbreak management, control, and prevention (43).

The present study did not evaluate the serum of both captive and free-living animals, primarily because of the difficulty of establishing specific conjugates for each animal species, as well as the high non-specificity of immunofluorescence conjugates used in wildlife investigations. Thus, currently available serological tests should be optimized and validated for each wildlife species before testing. Additionally, these species require specific handling, restraints, and anesthesia for safe sample collection. Nonetheless, future studies should include testing of these animals, particularly for acute and chronic symptoms, such as reproductive and respiratory disorders, which may be associated with Q fever. Such concurrent serological and molecular surveys involving wildlife professionals and the wildlife species they routinely handle could provide a One Health approach, determining whether exposure occurs and, if so, at what level of job position, location, and wildlife species contact. Finally, wildlife should always be considered as a potential source of *C. burnetii* transmission to both domestic animals (companion and livestock) and humans, particularly in areas that overlap with or are adjacent to wildlife habitats.

This study is the first serological investigation of *C. burnetii* in park rangers, zookeepers, and animal service workers in Brazil, with an overall seroprevalence of 8.1% and no associated risk factors for seropositivity. Given that the seroprevalence in this study was higher than that reported in previous surveys of healthy (asymptomatic) human populations, *C. burnetii* exposure may also represent an occupational risk for wildlife professionals in contact with natural environments in Brazil.

## Data Availability

The raw data supporting the conclusions of this article will be made available by the authors, without undue reservation.
